# Interventional Effects of Edible Bird's Nest or Sialic Acids on Sepsis‐Induced Immunosuppression in Mice

**DOI:** 10.1002/fsn3.71836

**Published:** 2026-05-18

**Authors:** Shentang Li, Yu Jiang, Ke Wang, Haidan Li, Jie Li, Jin Huang, Jia Wang, Lin Zhou, Yuanzhu Xie, Yanjuan Liu

**Affiliations:** ^1^ Department of Pediatrics, The Third Xiangya Hospital Central South University Changsha Hunan China; ^2^ Department of Emergency Medicine Hunan Provincial People's Hospital (The First Affiliated Hospital of Hunan Normal University) Changsha China; ^3^ The Second Xiangya Hospital of Central South University Changsha China; ^4^ Department of Respiratory and Critical Care Medicine Hunan Provincial People's Hospital/The First Affiliated Hospital of Hunan Normal University Changsha China; ^5^ College of Clinical Laboratory Changsha Medical University Changsha Hunan China

**Keywords:** Edible bird's nest, mTOR pathway, sepsis‐induced immunosuppression, sialic acid, T lymphocyte

## Abstract

Edible bird's nest (EBN) and its key component, sialic acid (SA), were investigated for their potential to counteract sepsis‐induced immunosuppression. In a murine “two‐hit” sepsis model, EBN and SA significantly enhanced survival, ameliorated multi‐organ damage, and reduced bacterial dissemination. Crucially, they reversed immunosuppression by restoring the TNF‐α‐secreting capacity of immune cells. Proteomic and KEGG analyses identified T cell proliferation as a central mechanism, which was validated by CFSE assay. Furthermore, we demonstrated that this enhanced T cell proliferation is mediated through the activation of the mTOR signaling pathway. This study reveals that EBN and SA are promising immune‐enhancing agents against sepsis‐induced immunosuppression, offering a novel therapeutic strategy for secondary infections.

## Graphical abstract

This study reveals that edible bird's nest (EBN) and sialic acid (SA) efectively reverse sepsis‐induced immunosuppression. EBN and SA enhance T cell proliferation via the activation of the mTOR signaling pathway, and this enhanced proliferation mediates the protective effect. These findings highlight the role of nutritional bioactive compounds in immune regulation and may inform future therapeutic strategies for secondary infections in sepsis.

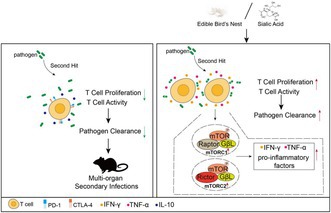

## 1 Introduction

Sepsis is a biphasic disorder characterized by an initial hyper‐inflammatory phase followed by prolonged immunosuppression (Venet and Monneret 2017). The immune response dynamics during sepsis progression are remarkably complex. Current evidence suggests that the immunosuppressive phase, characterized by predominant anti‐inflammatory cytokines release and impaired pro‐inflammatory cytokine secretion, contributes to approximately 85% of sepsis‐related mortality due to increased susceptibility to secondary infections (Nedeva et al. 2020). Despite extensive research, existing therapeutic strategies, including anti‐inflammatory interventions and immunoadjuvant therapies, have demonstrated limited clinical efficacy (Deinhardt‐Emmer et al. 2025). Consequently, the development of novel immunostimulatory agents represents a critical therapeutic approach for improving patient outcomes.

Edible Bird's Nest (EBN, Yan Wo) is a natural product composed of solidified saliva secreted by swiftlets ( *Aerodramus fuciphagus* and *Aerodramus maximus* ). Revered in Chinese culture since the Tang Dynasty, EBN is often termed the “caviar of the East” and has long been prized as a luxury health tonic (Kasem and Koon 1998). Traditional use attributes various therapeutic effects to EBN, including respiratory support, skin health enhancement, immune modulation, asthma relief, and improved skin complexion (Dai et al. 2021). Additionally, it has been associated with antiaging properties and accelerated wound healing (Hu et al. 2016). Asia countries are the main customer that widely used EBN as alternative medical foods and some European countries (Lai et al. 2022). In other words, the immune booster effect is still the dominant expectation of medical benefits of EBNs from the customer. However, despite these widely cited benefits of EBN, robust scientific validation in the context of sepsis‐induced immunosuppression remains lacking.

Sialic acid (SA), a group of acidic aminoglycans abundantly found in EBN, accounting for the content of about 14% (Qian et al. 2025), has garnered significant interest in medical and biological research (Bo et al. 2025; Kukan et al. 2025; Haghani et al. 2017). It is also known as “EBN acid” because of its highest content in EBN compared to other natural products. The SA family comprises multiple members, with N‐acetylneuraminic acid (Neu5Ac) and its derivatives being the most common (Ling et al. 2022). Structurally, SA consists of a nine‐carbon glycoside derivative formed through the condensation of pyruvic acid and mannosamine. Studies have demonstrated its crucial roles in anti‐oxidation (Pawluczyk et al. 2015), anti‐inflammation (Tian et al. 2025), antivirus (Haghani et al. 2017), antitumor (Vatankhah et al. 2025) and immune regulation (Li et al. 2024). Despite these findings, the mechanistic role of SA in sepsis‐induced immunosuppression remains insufficiently explored.

In light of these considerations, this study was designed to develop a mouse model of sepsis‐induced immunosuppression. Using EBN and SA, we investigated their effects on sepsis‐induced immunosuppression, focusing on the key parameters such as multi‐organ damage, T cell proliferation, and bacterial clearance efficiency. The results are anticipated to advance our understanding of the mechanisms by which SA and EBN improve immunosuppression in sepsis, thereby offering valuable insights for the development of novel therapeutic interventions.

## 2 Materials and Methods

### 2.1 Animals

Fifty male C57BL/6 mice (6–8 weeks old, weighing 20–25 g) purchased from Henan Sikebeisi Biotechnology Co. Ltd. (SCXK (Yu) 2020‐0005) were housed in the SPF‐grade animal facility of the Central Laboratory at the First Affiliated Hospital of Hunan Normal University (Hunan Provincial People's Hospital). All the animals underwent a 1‐week acclimatization period prior to experiments under controlled environmental conditions: temperature maintained at 22°C–28°C, relative humidity 40%–60%, 12/12 h light/dark cycle, noise level < 60 dB, ammonia concentration ≤ 20 ppm, and air exchange rate of 16 times/h, with all procedures strictly following the animal care guidelines approved by the Hunan Provincial People's Hospital/The First Affiliated Hospital of Hunan Normal University Institutional Animal Ethics Committee.

### 2.2 Preparation of EBN Extract

White EBN, sourced from a commercial production facility in Malaysia, was procured through Chinese market channels. The raw material was maintained under ambient storage conditions prior to processing. For extraction, EBN samples were precisely weighed and subjected to overnight hydration in double deionized (DDI) water at a 1:100 (w/v) ratio. Following hydration, the material underwent three successive rinses with DDI water to eliminate inorganic contaminants. The cleaned EBN was then subjected to thermal extraction in DDI water (1:30, w/v) at 98°C ± 2°C for 8 h with continuous mechanical agitation, yielding the final EBN extract (Yew et al. 2019).

### 2.3 Sepsis Animal Model Established

All animals underwent an 8‐h fasting period and 4‐h water deprivation prior to cecal ligation and puncture (CLP) surgery. Surgical procedures were performed under continuous 2% isoflurane anesthesia. Following a 1 cm midline abdominal incision, the cecum was carefully exposed. Approximately 75% of the cecal length was ligated with 4‐0 silk suture to ensure uniform content distribution, followed by double puncture with a 21‐gauge needle (Yan et al. 2026).

### 2.4 “Two‐Hit” Sepsis Animal Model Construction and EBN Treatment

The septic mouse model was established as described above (Shiwei et al. 2012). Three days after surgery, the mice received daily administrations via oral gavage of either 200 mg/kg of EBN or 28 mg/kg of SA (Aladdin, NO: A100555), which continued for 15 consecutive days, with the dosage based on a previously reported protocol (Qian et al. 2025). On postoperative day 14, *Pseudomonas aeruginosa* (P.a, 16 × 10^9^ CFU/10 g) was administered via intratracheal instillation. Following inoculation, the mice were kept in a warm environment for 30 min before being transferred back to the animal facility for standard housing. Survival rates were recorded at 15 days after CLP surgery.

### 2.5 Hematoxylin–Eosin (H&E) Stain and Bacterial Clearance Assay

Mice were euthanized, and the lung, liver, and kidney were aseptically collected. A precisely weighed portion (100 mg) of each tissue was homogenized in 1 mL of sterile PBS using a tissue homogenizer. The homogenate was then serially diluted 10‐fold in sterile PBS. From appropriate dilutions, 100 μL aliquots were plated onto Luria‐Bertani (LB) agar plates. The plates were incubated at 37°C for 24 h. Following incubation, bacterial colonies were counted manually. Another portion was snap‐frozen in liquid nitrogen and stored at −80°C. The remaining samples were fixed in 4% formaldehyde for 24 h following standard protocols (Zhang et al. 2019). After paraffin embedding and sectioning (5 μm thickness), tissue morphology was assessed by H&E staining.

### 2.6 Serum Collection

At postoperative days 14, mice were anesthetized using 2% isoflurane and blood samples were obtained via cardiac puncture. Serum was separated by centrifugation and stored at −80°C for subsequent biochemical analysis and inflammatory cytokine quantification, with the general experimental steps referring to protocol (Aurbach et al. 2019).

### 2.7 Proteomic Analysis

Proteomic analysis was performed as previously described (Xiao et al. 2024). Splenic samples stored in liquid nitrogen were cryogenically ground into powder using a Tissuelyser‐24 homogenizer (Shanghai Jingxin) with four 1‐min liquid nitrogen cycles. Proteins were extracted by adding 1× SDS lysis buffer followed by 30‐min rotation at 4°C, then subjected to pulsed ultrasonication (8 s on/8 s off for 10 min at 4°C) using a Scientz08‐III homogenizer (Ningbo Scientz). After centrifugation (12,000 g, 20 min), supernatants were collected and quantified using a MicroBCA kit (Thermo Fisher). For MS analysis, proteins were acetone‐precipitated, urea‐dissolved, and trypsin‐digested into peptides. LC–MS/MS was performed on a NanoAcquity UHPLC‐Orbitrap Exploris 240 system (Thermo Fisher) in DIA mode. 0.5 μg peptides were trapped (10 μL/min, 4 min) and separated on a C18 column (75 μm × 250 mm, 2 μm) at 300 nL/min over 150 min with the following gradient: 0–3 min: 3% B; 3–5 min: 3%–5% B; 5–125 min: 5%–40% B, 125–135 min: 40%–95% B, 135–139 min: 95% B; 139–140 min: 95%–3% B; 140–150 min: 3% B. Buffer A contained 0.1% Formic acid (FA) in HPLC water, while buffer B contained 80% acetonitrile (CAN) and 0.1% FA in HPLC water. MS parameters included: 2.1 kV spray voltage, 300°C capillary temperature, full MS (350–1200 m/z, 6000 resolution), and DIA scans (10 m/z windows, 15,000 resolution, 30% CE). Data were processed using DIA‐NN (v1.8.1) against UniProt human proteome (2022‐10‐20) with 5% FDR, and DEPs were identified using DEP packages (v1.16.0).

### 2.8 Isolation of Splenic Lymphocytes

At day 14 post‐CLP, mice were euthanized and spleens were aseptically harvested. The organs were mechanically dissociated and passed through a 70 μm cell strainer to obtain single‐cell suspensions. Lymphocytes were then isolated by Ficoll–Hypaque density gradient centrifugation (GE Healthcare, USA), washed twice with PBS, and prepared for subsequent flow cytometry analysis (Anyalebechi et al. 2024).

### 2.9 Flow Cytometry Analysis

The expression levels of CD4, CD8, TNF‐α, CD279 (PD‐1), and CD152 (CTLA‐4) were evaluated by flow cytometry, with intracellular TNF‐α detection performed using a fixation/permeabilization kit (BD Biosciences, Cat: 2220750). Briefly, splenic lymphocytes were isolated and washed with PBS three times. Then, the cells were resuspended in 1 mL PBS, counted by light microscopy, and adjusted to 1 × 10^6^ cells/mL. Cell aliquots were stained with BV‐421‐anti‐TNF‐α (Invitrogen, LOT: 2763919), PerCP‐Cy5.5‐anti‐CD8 (BD Biosciences, CAT: 551162), PE‐Cy7‐anti‐CD279 (Invitrogen, LOT: 2050427), Alexa Fluor 700‐anti‐CD4 (BD Biosciences, CAT: 557956), and PE‐anti‐CD152 (Invitrogen, LOT: 3016421) antibodies for 30 min at 4°C before acquisition on a BD FACS Canto II flow cytometer and subsequent analysis using FlowJo software (Version X; TreeStar) (Abdelhafiz et al. 2025).

### 2.10 Assessment of T Cell Proliferation

CFSE, a widely used fluorescent dye for tracking cell proliferation, was employed according to the manufacturer's protocol. Briefly, cells were resuspended in PBS (1 × 10^6^ cells/mL) and labeled with 10 μM CFSE (Yeasen biotech Co. Ltd., Shanghai, China, NO: 40714ES76) for 10 min at 37°C. The reaction was stopped by adding five volumes of ice‐cold complete medium, followed by a 5 min incubation on ice. After centrifugation, cells were washed three times with fresh medium, cultured for 24 h, and analyzed by flow cytometry (Lyons 2000).

### 2.11 Western Blot Analysis

CD3^+^ T cells were purified by magnetic separation using anti‐CD3 beads (Miltenyi Biotec, Bergisch Gladbach, Germany) according to the manufacturer's instructions. Purified CD3^+^ T cells were isolated for western blot analysis. Firstly, proteins from CD3^+^ T cells were separated by 10% SDS‐PAGE and transferred to PVDF membranes. After blocking for 1 h at room temperature, the membranes were incubated overnight at 4°C with primary antibodies against GβL (CST, Cat NO: 9964T), mTOR (CST, Cat NO: 9964T), p‐mTOR (CST, Cat NO: 9964T), raptor (CST, Cat NO: 9964T), rictor (CST, Cat NO: 9964T), and β‐Actin (CST, Cat NO: 4970S). Following three 10‐min washes, the blots were probed with HRP‐conjugated goat anti‐rabbit IgG for 1 h at room temperature, and protein bands were visualized using ECL reagents (Yan et al. 2026).

### 2.12 LC–MS/MS

The chemical profiling of EBN was performed using ultra‐high‐performance liquid chromatography coupled with tandem mass spectrometry. Specifically, a Vanquish UHPLC system (Thermo Fisher Scientific) equipped with a Phenomenex Kinetex C18 column (2.1 mm × 100 mm, 2.6 μm) was employed for separation, with the mobile phase consisting of 0.01% acetic acid in water (A) and isopropanol/acetonitrile (1:1, v/v) (B). The autosampler temperature was maintained at 4°C, and the injection volume was set to 2 μL. Mass spectrometric detection was conducted on an Orbitrap Exploris 120 instrument (Thermo Fisher Scientific) operating in data‐dependent acquisition (DDA) mode under Xcalibur software control. The ESI source parameters were as follows: sheath gas, 50 Arb; auxiliary gas, 15 Arb; capillary temperature, 320°C; full MS resolution, 60,000; MS/MS resolution, 15,000; stepped NCE, 20/30/40. Spray voltages were optimized at 3.8 kV for positive ion mode and −3.4 kV for negative ion mode, according to the previously established protocol (Dunn et al. 2011).

### 2.13 Statistical Analysis

All data were presented with mean ± SD and statistic analyzed by GraphPad Prism 9.0 software. The Student's unpaired *t*‐test was used to determine statistical significance between the two groups. Two‐way analysis of variance was applied to compare the significant difference among multiple groups. *p* < 0.05 was considered statistically significant.

## 3 Result

### 3.1 Mass Spectrometric Identification of SA in EBN

**FIGURE 1:**
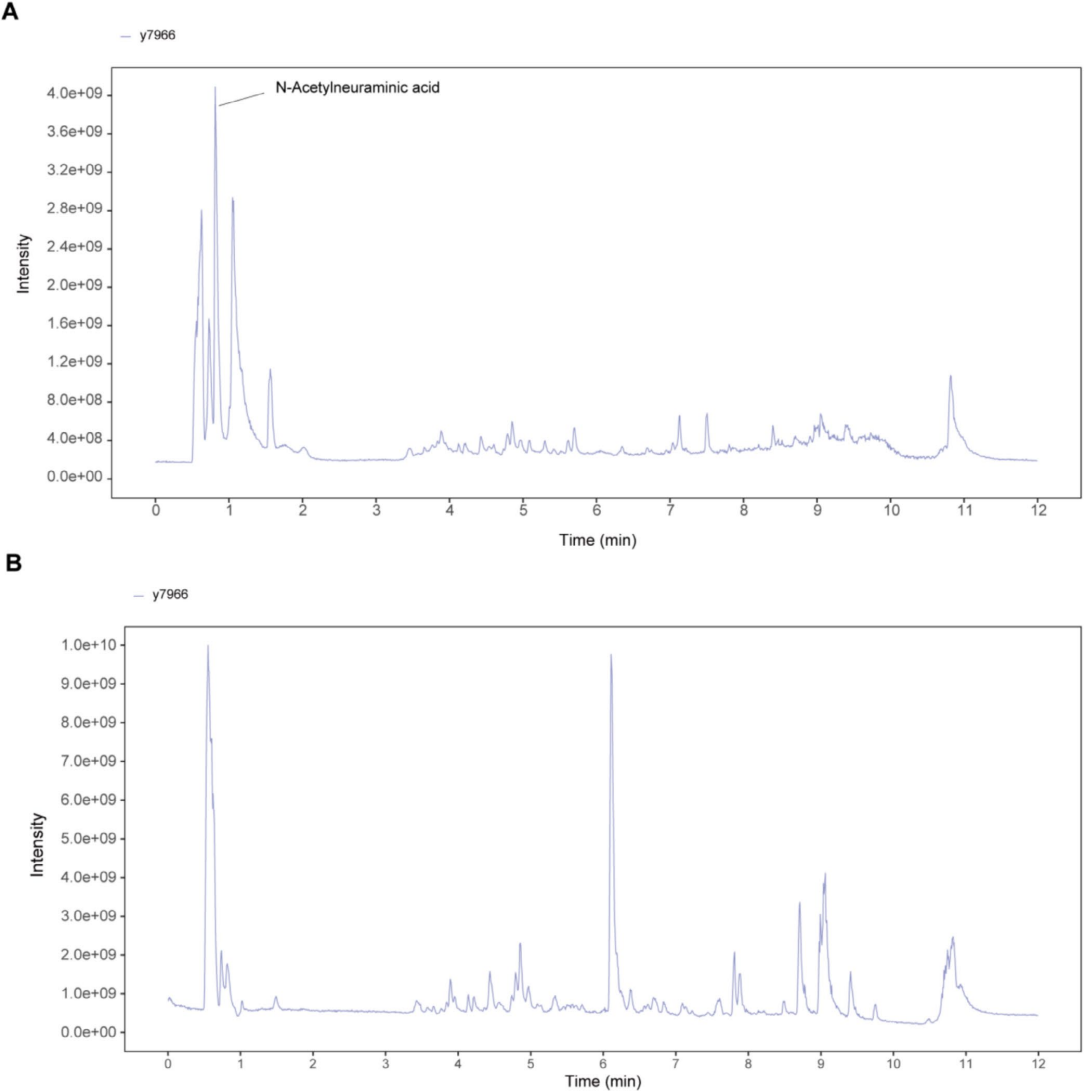
Identification of SA as a major component of EBN. (A) MS spectrum of EBN in negative ion mode. (B) MS spectrum of EBN in positive ion mode.

The chemical constituents of Edible Bird's Nest (EBN) were characterized by LC–MS/MS. Anion and cation profiling identified a total of 476 major compounds, including sialic acid (SA), various proteins, and glycoproteins (Table S1). Notably, SA was determined to be one of the principal components of EBN (Figure 1A).

### 3.2 EBN or SA Exerted Protective Effects Against Multiple Organ Dysfunction and Exhibited Immunomodulatory Activity in a “Two‐Hit” Sepsis Murine Model

**FIGURE 2:**
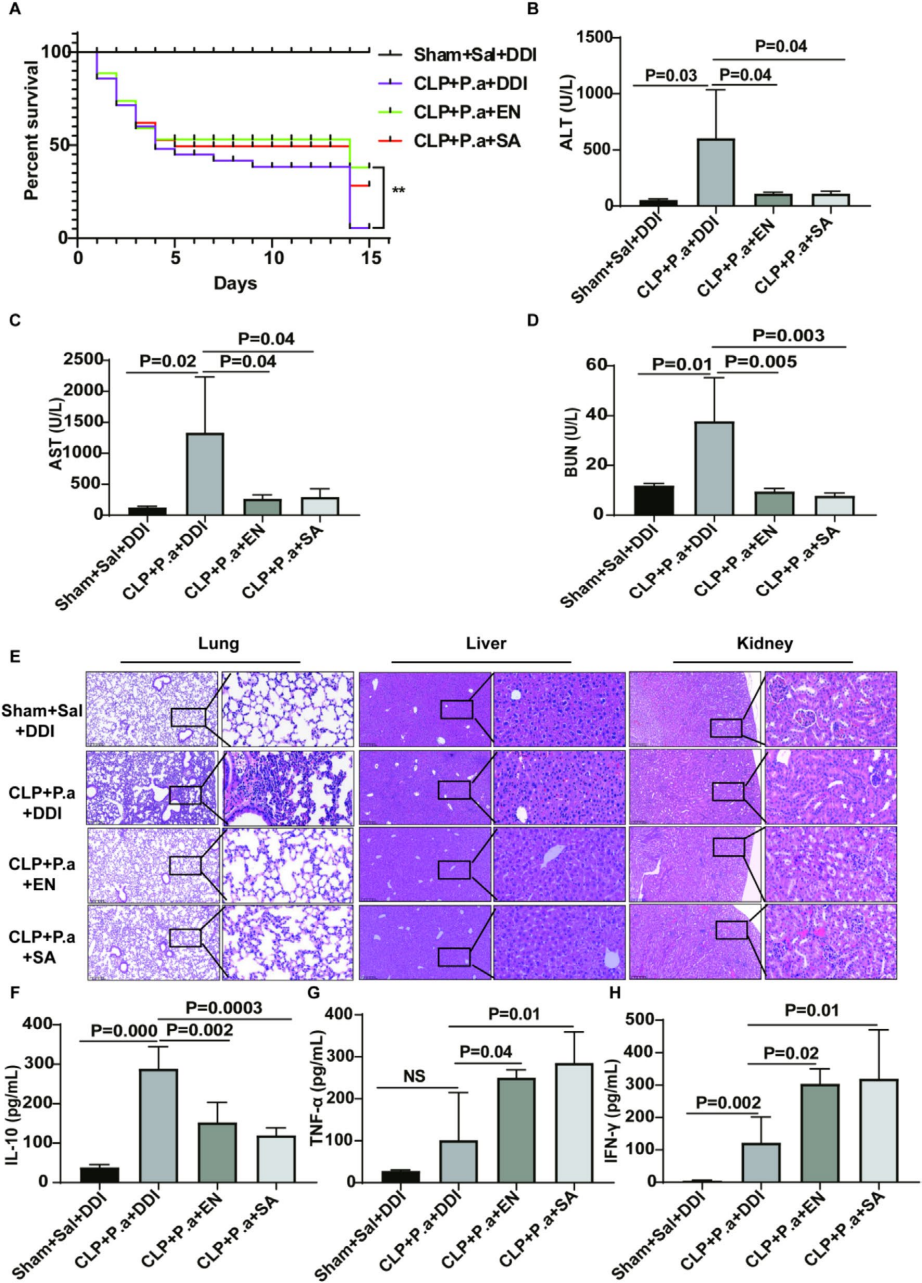
EBN or SA exerted protective effects against multiple organ dysfunction and exhibited immunomodulatory activity. (A) EBN or SA treatment improved the survival rate of sepsis mice (*n* = 20). (B–D) ALT, AST, and BUN contents in EBN‐ or SA‐treated mice using biochemical analysis (*n* = 6). (E) EBN or SA alleviated the multiple organ injury induced by sepsis using H&E staining (*n* = 4). (F) Anti‐inflammatory cytokines contents in EBN‐ or SA‐treated mice using biochemical analysis (*n* = 6). (G, H) Pro‐inflammatory cytokines contents in SJS‐treated mice using biochemical analysis (*n* = 6).

The protective effects of SA and EBN on the survival rate, organ function and immunomodulatory effects were shown in Figure 1. Compared with the Sham+Sal+DDI group, more C57BL/6 mice died in the CLP + P.a + DDI group, whereas the EBN or SA‐treated mice showed a strong decrease. We observed that prior to the secondary challenge on day 14, survival rates in both the DDI‐gavaged group and the other groups were similar (approximately 50%). However, following *Pseudomonas aeruginosa* infection, the DDI‐gavaged group showed significantly higher mortality compared to the EBN‐treated or SA‐treated groups (Figure 2A). Besides, the liver and kidney function became significantly more prominent in the CLP + P.a + DDI group compared with the Sham+Sal+DDI group (Figure 2B–D). Among them, the EBN or SA‐treated group showed a significant elevation in the kidney and liver function compared to the CLP + P.a + DDI group (Figure 2B–D). HE staining results were highly similar to those obtained by biochemical analysis. Additionally, our ELISA data showed that EBN or SA acted to modulate the cytokines release and boost immune response, evidenced by promoting the production of pro‐inflammatory cytokines and suppressing the anti‐inflammatory cytokines release (Figure 2F–H).

### 3.3 EBN or SA Enhanced Bacterial Clearance in a “Two‐Hit” Sepsis Murine Model

**FIGURE 3:**
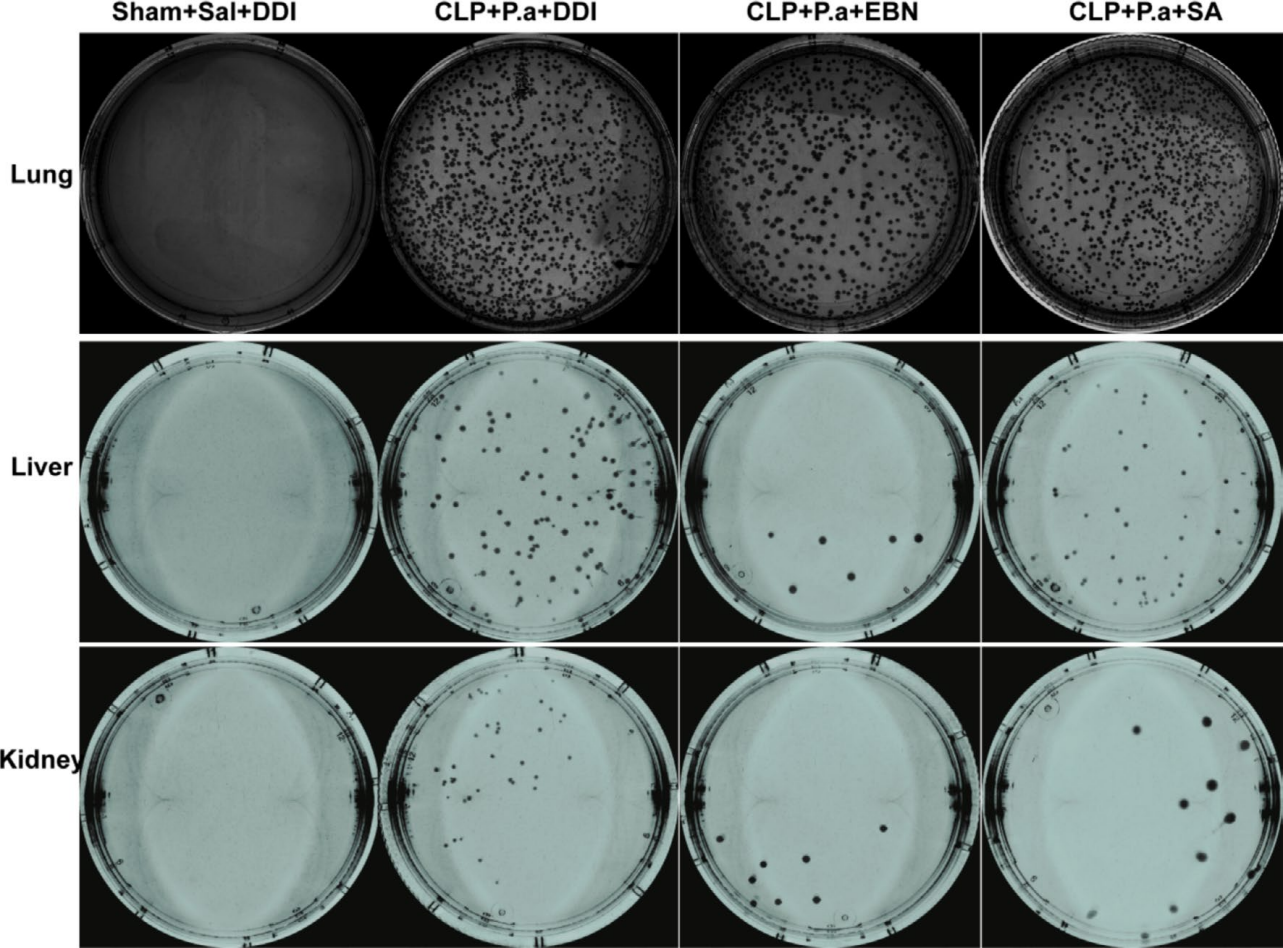
EBN or SA enhanced bacterial clearance in a “two‐hit” sepsis murine model (*n* = 5).

Previous studies have established that immunosuppressed states markedly compromise bacterial clearance ability. To investigate this phenomenon, we intratracheally challenged mice with *Pseudomonas aeruginosa* on postoperative day 13 following CLP surgery. Quantitative analysis of bacterial burden in pulmonary, splenic, and renal tissues 24 h postinfection revealed striking differences. No cultivable bacteria were detected in Sham+Sal+DDI controls, while the CLP + P.a + DDI group showed extensive P.a dissemination across all examined organs (Figure 3). Notably, therapeutic intervention with either EBN or SA resulted in statistically significant reductions in bacterial colonization compared to untreated septic mice (CLP + P.a + DDI) (Figure 3). These data collectively demonstrate that both EBN and SA possess immunopotentiating properties capable of restoring antimicrobial defense mechanisms in immunocompromised hosts.

### 3.4 Proteomics Analyses of Spleens

**FIGURE 4:**
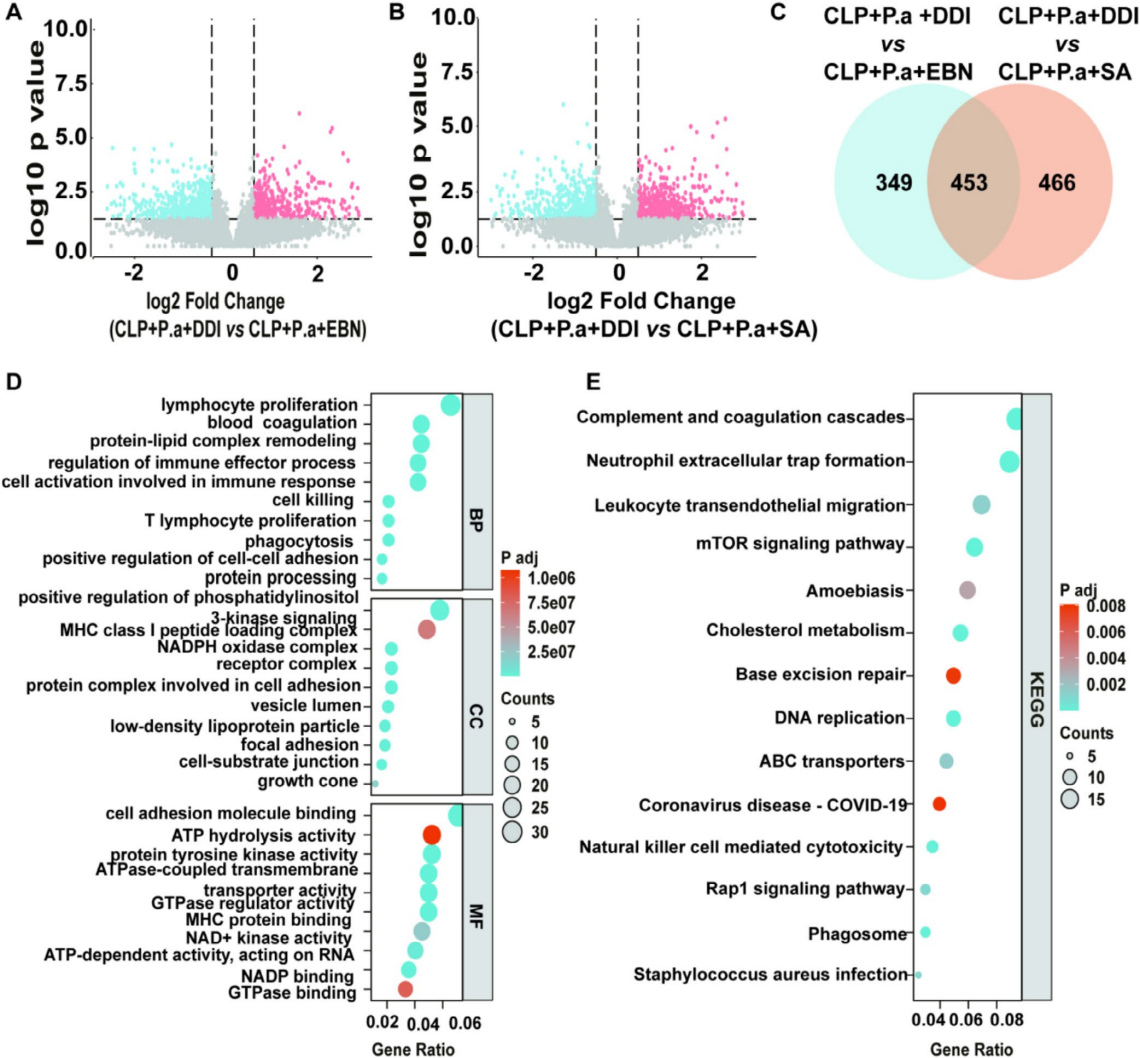
Proteomic analysis. (A) DEPs between the CLP + P.a + DDI and CLP + P.a + EBN group (*n* = 4). (B) DEPs between the CLP + P.a + DDI and CLP + P.a + SA group (*n* = 4). (C) Venn diagram of the DEPs. (D, E) GO and KEGG analysis of the shared DEPs.

To investigate the underlying mechanisms by which EBN or SA modulates the immune response, we performed proteomics analyses. As shown in Figure 3, the CLP + P.a + EBN group exhibited 988 differentially expressed proteins (DEPs), including 419 upregulated and 569 downregulated, compared to the CLP + P.a + DDI group (Figure 4A). Furthermore, the CLP + P.a + SA group showed 919 DEPs, containing 498 upregulated and 421 downregulated, when compared to the CLP + P.a + DDI group (Figure 4B). Our Venn diagram identified 453 shared DEPs (Figure 4C). GO analysis showed these common DEPs were enriched in cell proliferation, immune response, and energy modulation (Figure 4D). KEGG pathway analysis showed these common DEPs were highly enriched in the mTOR signaling pathway (Figure 4E).

### 3.5 EBN or SA Improved Sepsis‐Induced Immunosuppression

**FIGURE 5:**
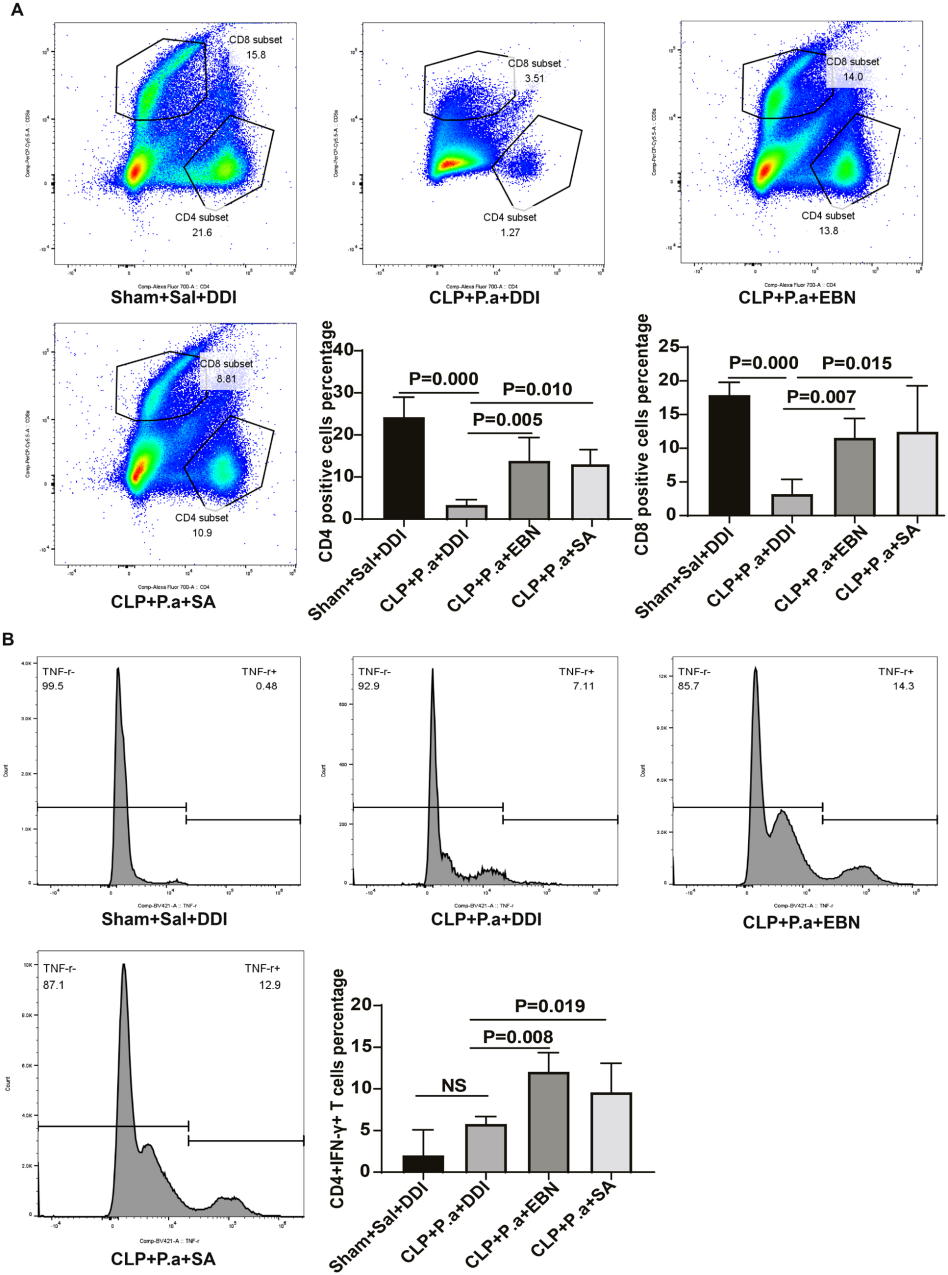
EBN or SA increased T cell proportions and TNF‐α secreting capability. (A) EBN and SA increased the proportion of CD4^+^ and CD8^+^ T cells. (A) EBN or SA increased the proportion of CD4^+^ and CD8^+^ T cell subsets (*n* = 6). (B) EBN or SA enhanced the T cell TNF‐α‐secreting capability (*n* = 6).

**FIGURE 6:**
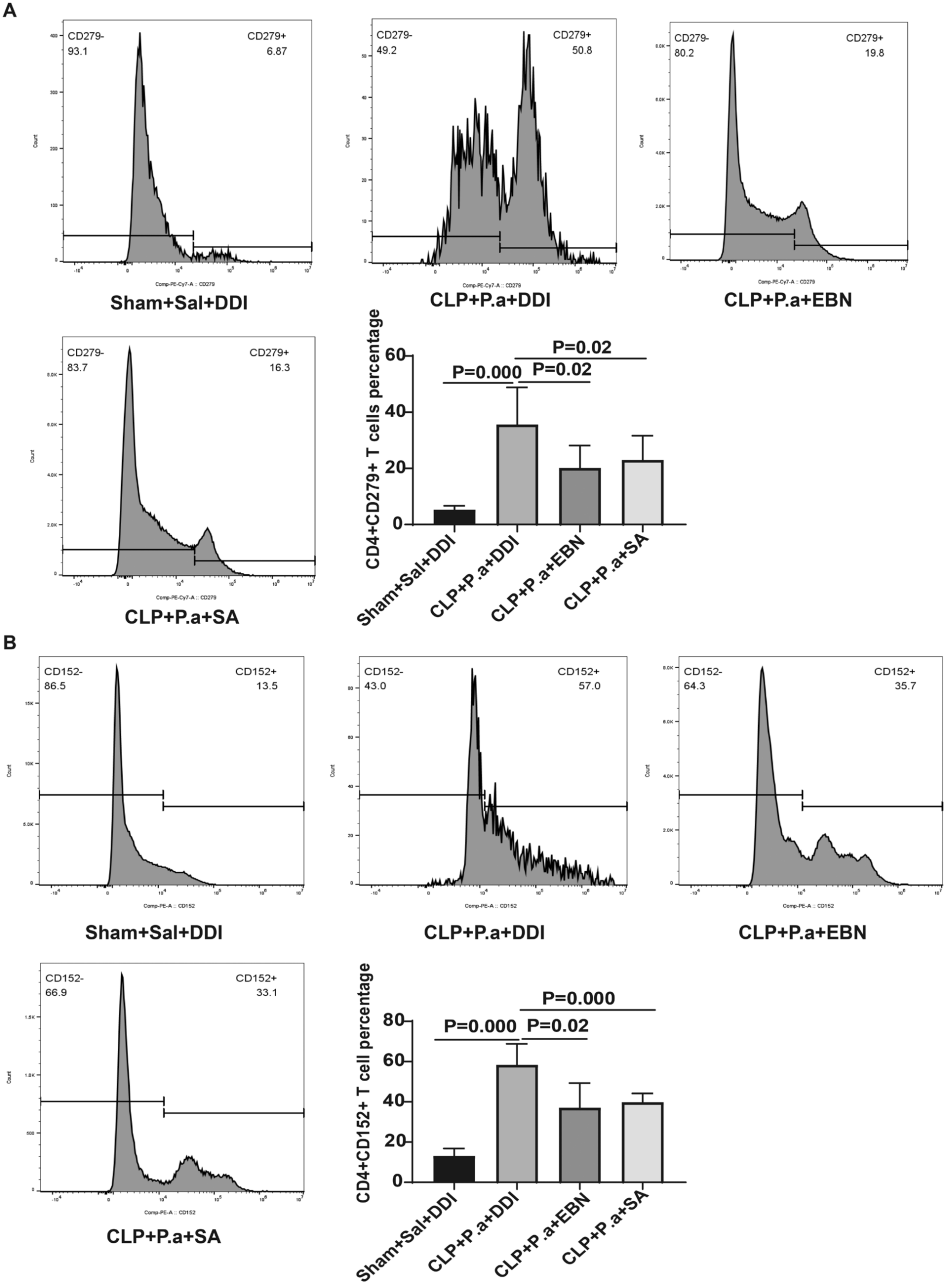
EBN or SA inhibited the expression of immune checkpoint molecules. (A, B) PD‐1 (A) and CTLA‐4 (B) expression in splenic T cells isolated from EBN‐ or SA‐treated septic mice (*n* = 6).

Sepsis‐induced immunosuppression is characterized by lymphopenia, upregulation of immune checkpoint molecules, and impaired TNF‐α production. To investigate the effects of EBN and SA on sepsis‐induced immunosuppression, we evaluated their effects on the proportion of T cell populations, TNF‐α secretion, and immune checkpoint molecule expression. Our results demonstrated that septic mice receiving a “second hit” showed significant reductions in both CD4^+^ and CD8^+^ T cell proportions (Figure 5A) and decreased TNF‐α levels (Figure 5B). However, treatment with either EBN or SA effectively restored T lymphocyte populations and enhanced TNF‐α production (Figure 5A,B). Flow cytometry analysis further revealed that the CLP + P.a + PBS group displayed substantial upregulation of PD‐1 (Figure 6A) and CTLA‐4 (Figure 6B), while EBN or SA administration significantly attenuated this expression. These findings collectively indicate that EBN or SA may counteract sepsis‐induced immunosuppression through reconstituting T cell homeostasis, augmenting pro‐inflammatory cytokine release, and suppressing immune checkpoint overexpression.

### 3.6 EBN or SA Promoted the Proliferation of T Cells

**FIGURE 7:**
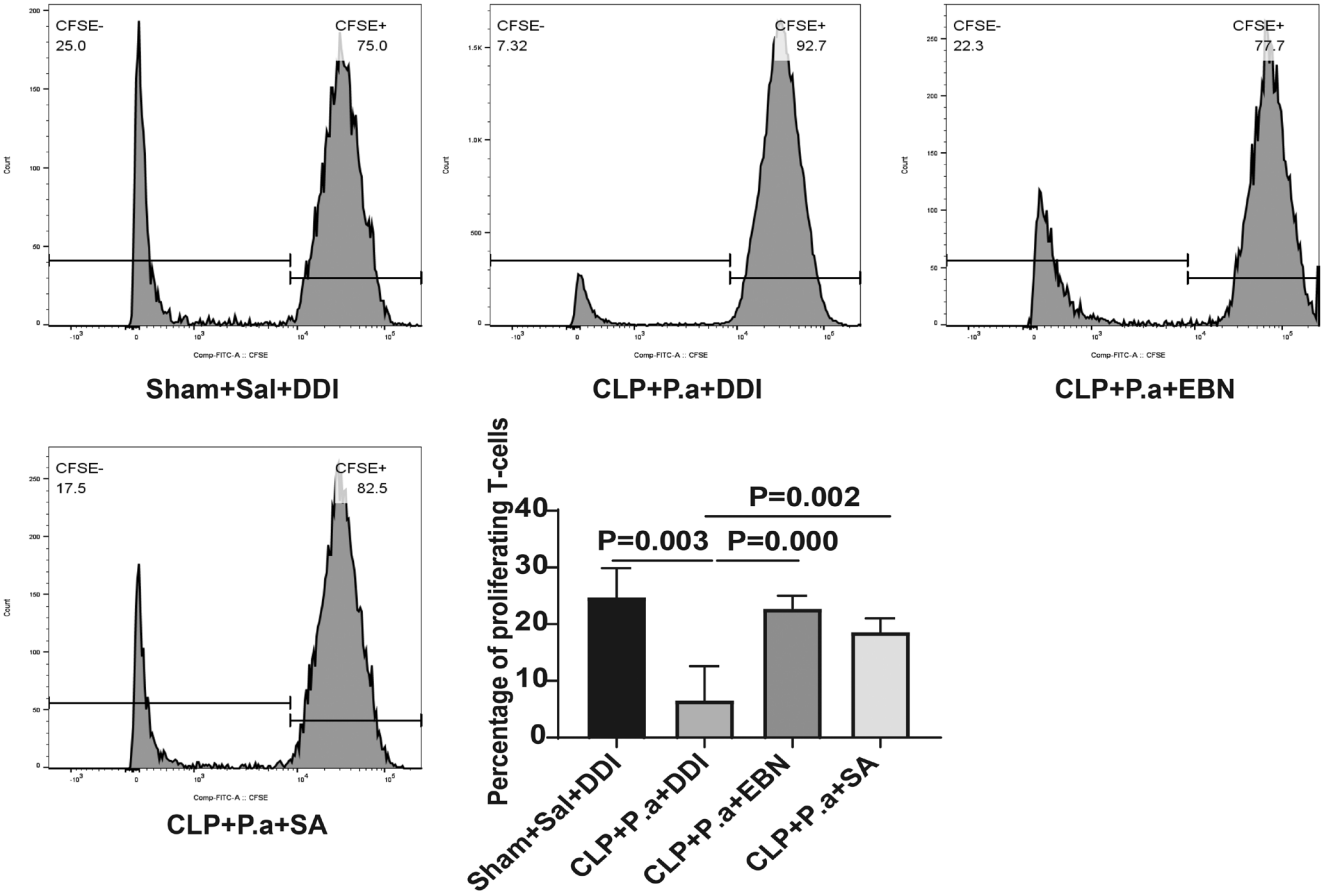
CFSE assay was performed to detect the proliferation of splenic T cells treated with EBN or SA (*n* = 6).

Our KEGG pathway analysis identified T cell proliferation as the primary pathway through which EBN and SA exert their effects in sepsis‐induced immunosuppression. To validate these findings, we assessed T cell proliferation using CFSE dilution assays. Our results showed that mice subjected to a “second hit” exhibited significantly impaired T cell proliferative capacity. Notably, both EBN and SA treatment partially restored this proliferation defect (Figure 7). These findings demonstrate that EBN and SA can effectively enhance T cell proliferation in the context of sepsis‐induced immunosuppression.

### 3.7 EBN or SA Modulated the Expression of Related Proteins in mTOR Pathway

**FIGURE 8:**
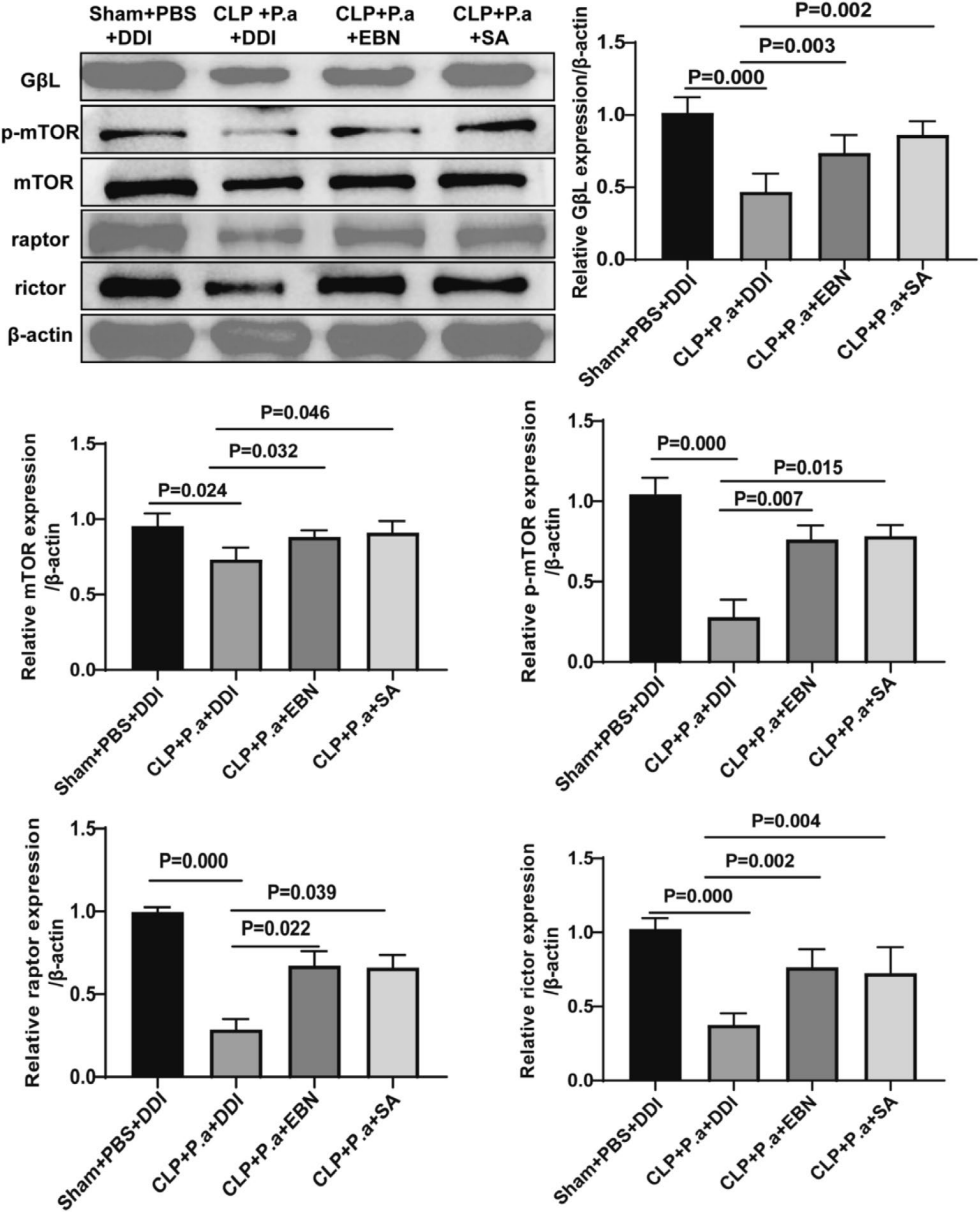
Western blot was performed to detect the proteins involved in the mTOR signaling pathway (*n* = 6).

Our proteomic analysis revealed the mTOR pathway was the key pathway EBN or SA exerted in sepsis‐induced immunosuppression, a pathway closely associated with cellular proliferation. Then, we isolated splenic T lymphocytes and examined the expression levels of the crucial proteins, such as GβL, phosphorylated mTOR (p‐mTOR), raptor, rictor in the mTOR pathway. Our western blot analysis showed significantly reduced levels of p‐mTOR, rictor, raptor and GβL in the CLP + P.a + DDI group compared to the controls (Figure 8). Notably, EBN or SA treatment partially restored the expression of these signaling molecules (Figure 8).

## 4 Discussion

Persistent sepsis‐induced immunosuppression remains a critical determinant of adverse outcomes, positioning immunostimulation as a rational therapeutic strategy (Gao et al. 2025; Mayr et al. 2013). Although various immunostimulants, such as thymosin α1 and other investigational adjuvants, have undergone clinical evaluation, few have demonstrated satisfactory efficacy, highlighting an unmet therapeutic need (Gao et al. 2025). In the current work, we found that EBN effectively accelerated the proliferation of T cells. We also observed improved immunity, based on the increased bacteria clearance and TNF‐α production in EBN‐ or SA‐treated septic mice. Simultaneously, we observed that the administration of either EBN or SA did not induce an excessive inflammatory response. Hence, we demonstrated that EBN or SA can reduce sepsis‐induced immunosuppression by facilitating the proliferation of T lymphocytes and enhancing these cells' TNF‐α‐secreting function. Our work thus paves the way for developing food‐derived interventions against sepsis and suggests a novel clinical repurposing of EBN and SA.

EBN demonstrates multifaceted biological activities, such as epidermal growth factor‐like properties (Zhang et al. 2012), antiviral effects (Haghani et al. 2016), and cellular proliferation (Zhao et al. 2016). Recognized as an essential energy substrate for cellular function and immune enhancement, its immunomodulatory capacity is well‐documented (Chua and Zukefli 2016). Zhao et al. demonstrated its reversal of drug‐induced immunosuppression via B‐cell activation and antibody potentiation (Zhao et al. 2016). Further studies confirm EBN can improve the morphology of secondary lymphoid organs and enhance the immune cells in both specific and nonspecific immune functions (Dobutr et al. 2023). In the current work, we demonstrated that EBN treatment effectively mitigated sepsis‐induced multi‐organ dysfunction and enhanced bacterial clearance capacity. Numerical studies have established that sepsis‐induced immunosuppression is characterized by T lymphopenia, elevated expression of immune checkpoint molecules (PD‐1 and CTLA‐4) (Wang et al. 2025; Venet and Monneret 2017). In addition, some researches reported during the late sepsis phase, both CD4^+^ and CD8^+^ T cells develop an exhausted phenotype, exhibiting reduced proliferative capacity and diminished TNF‐α secretion (Qin et al. 2024), a key cytokine that enhances monocyte antigen presentation and phagocytic activity (Vámos et al. 2025). Notably, the upregulation of immune checkpoint molecules directly contributes to T cell dysfunction, as evidenced by functional recovery following their blockade (Hotchkiss et al. 2019). Our data showed EBN functioned to promote T cell proliferation, enhance TNF‐α secreting capability and inhibit co‐inhibitory receptors expression, suggesting its therapeutic potential in reversing sepsis‐associated immunosuppression.

Sialic acids (SA), predominantly N‐acetylneuraminic acid (Neu5Ac) in humans, are ubiquitously expressed on immune cell surfaces, where they modulate cell–cell interactions and signaling pathways critical for immune activation. Beyond their role as cell surface components, exogenous SA supplementation has been shown to modulate immune cell functions (Qian et al. 2025). In particular, dietary or pharmacologically administered SA can influence T cell responses, including effects on T cell receptor signaling and proliferation. As crucial glycocalyx components, SA orchestrate immune recognition and response through their interactions with sialic acid‐binding immunoglobulin‐type lectins (Siglecs) (Radu and Baek 2025). These interactions modulate both innate and adaptive immunity, with emerging evidence underscoring Siglecs' pivotal roles in sepsis pathogenesis and therapeutic potential. The interaction between SA and Siglecs constitutes a key axis of immune regulation, capable of both dampening and enhancing immune responses depending on the specific Siglec subtype engaged and the cellular context. Specifically, Siglec‐1, ‐5, and ‐14 demonstrate bidirectional regulatory effects in sepsis by fine‐tuning inflammatory and immune responses (Liu et al. 2017), while Siglec‐2 (CD22) maintains immune homeostasis through B‐cell and T‐cell activation during infection (Yuan et al. 2025; Liu et al. 2025). Additionally, Siglec‐7 promotes monocyte‐mediated inflammation by upregulating pro‐inflammatory cytokines and chemokines via ERK pathway activation following pathogen recognition (Varchetta et al. 2012). Collectively, these findings establish SA as an active immunomodulator rather than merely a structural glycocalyx component. Our study revealed that SA enhances immune responsiveness by stimulating T‐cell proliferation through mTOR signaling pathway activation, which adds to the growing understanding of SA as a bioactive molecule capable of directly influencing lymphocyte function, complementing the well‐established regulatory mechanisms.

The mammalian target of rapamycin (mTOR) signaling pathway regulates diverse cellular processes, including apoptosis, autophagy, translation, and energy metabolism (Soltani et al. 2017). In mammals, mTOR, a highly conserved serine/threonine protein kinase, interacts with multiple proteins to form two distinct multiprotein complexes, namely mTOR complex 1 (mTORC1) and mTOR complex 2 (mTORC2). mTORC1, a rapamycin‐sensitive complex, consists of mTOR bound to regulatory‐associated protein of mTOR (raptor) and mammalian lethal with Sec13 protein 8 (GβL). Similarly, mTORC2 contains GβL but substitutes raptor with two unique components, including stress‐activated MAP kinase‐interacting protein 1 (mSin1) and rapamycin‐insensitive companion of mTOR (rictor) (Bao et al. 2025). At the molecular level, mTOR can modulate the activity of the downstream signals, such as elF4G and 4E‐BP1/2, thus promoting cellular proliferation (Khanal et al. 2025). In our study, we observed that EBN or SA partially restored the expression of GβL, p‐mTOR, rictor, and raptor, suggesting their ability to modulate both mTORC1 and mTORC2 complexes. Nevertheless, further investigation is necessary to determine whether SA alone can fully replicate the broad therapeutic effects mediated by EBN's complex bioactive composition.

The exclusive reliance on a mouse model constrains direct clinical translation due to known immunological differences between species. A key step toward improving translational relevance is the calculation of human equivalent doses (HED) based on body surface area normalization, which provides a rational basis for selecting safe starting doses in human studies. Beyond dose translation, potential variability in the bioactive composition of EBN from different natural sources may affect reproducibility. To address these limitations, future research should incorporate human immune cell models, establish HED‐validated dosing regimens, and employ rigorously standardized EBN preparations with quantified sialic acid content to ensure consistent biological activity in translational studies.

## 5 Conclusion

In conclusion, our study demonstrates, for the first time, that EBN and its major active component, SA, improve sepsis prognosis and attenuate multi‐organ injury in a murine model. Furthermore, we reveal that EBN and SA exert protective effects against sepsis‐induced immunosuppression, primarily by elevating TNF‐α levels and enhancing T‐cell proliferation. While these findings highlight SA as a key immunomodulatory constituent of EBN, further research is needed to determine whether SA alone can fully replicate the comprehensive therapeutic benefits of EBN. Collectively, our results support the potential of EBN as a therapeutic intervention for preventing immunosuppression in sepsis.

## Author Contributions

**Shentang Li:** data curation, writing – original draft. **Yu Jiang:** data curation, software. **Ke Wang:** data curation, software. **Haidan Li:** data curation, investigation. **Jie Li:** data curation. **Jin Huang:** data curation. **Jia Wang:** data curation, funding acquisition. **Lin Zhou:** methodology. **Yuanzhu Xie:** writing – original draft, funding acquisition. **Yanjuan Liu:** funding acquisition, data curation, writing – original draft, project administration.

## Funding

This project was financially supported by the National Natural Science Foundation of China (82102278), the Natural Science Foundation of Hunan Province (2024JJ8203, 2024JJ6275), Key Research Project of Health Commission of Hunan Province (20255386), the Scientific Research Project of Hunan Provincial Health Commission (W20243096), the Natural Science Foundation of Changsha City (kq2403138), Research Project of Hunan Provincial Department of Education (24B0090), Hunan Provincial Natural Science Foundation‐Enterprise Joint Project (2025JJ90189), the Key Cultivation Project of Hunan Provincial People's Hospital (RS2022A08) and Hunan Provincial Health High‐Level Talent Scientific Research Project (R2023018).

## Ethics Statement

The study protocol was approved by the Ethics Committee of Hunan Provincial People's Hospital (The First Affiliated Hospital of Hunan Normal University) (Approval No. 2021098) on January 5, 2022.

## Conflicts of Interest

The authors declare no conflicts of interest.

## Supporting information


**Table S1:** LC–MS/MS characterization of the chemical constituents of edible bird's nest (EBN).

## Data Availability

The data that support the findings of this study are available from the corresponding author upon reasonable request.
